# Medical Reconstruction and Graduate Teaching

**Published:** 1918-12-14

**Authors:** 


### The Hospital

The Workers' Newspaper of Administrative Medicine and Institutional Life, Administration, National Insurance and Health.

No. 1697, Vol. LXV. SATURDAY, DECEMBER 14, 1918. PRICE TWOPENCE

### MEDICAL RECONSTRUCTION AND GRADUATE TEACHING.

The repeated discussions on the subject of medical reconstruction which have marked the course of the last few years must be taken to mean a fairly widespread conviction that all is not well in the medical world. The discontent so indicated is no bad symptom and is certainly better than a dull satisfaction with existing conditions. It is especially worthy of note that on none of the many occasions which have presented themselves has any speaker appeared as a defender of things as they are or has urged the plaintive question: "Why can't you let it alone?" Reform and reconstruction are in the air, and everyone seems to agree that something radical must be undertaken. Medicine is often reproached with an undue inclination towards a conservative attitude, but just now every proclaimed reformer commands a ready audience. The future will show whether the profession can command the leader and the loyalty necessary to translate this prolonged debate into constructive action.

In most of the discussions on this topic the most exciting point has been found in the question of a proposed State Medical Service, and both advocates and opponents of the proposal have shown themselves men of strong conviction and earnest faith. To make any such scheme effective legislation would, of course, be necessary, and the enterprise may fairly be described as reform from without. There is, however, an obvious possibility of reform from within, and suggestions in this direction have by no means been wanting. The latest group of these may be found fairly well represented in the recent discussion at the Medical Society of London. The Society has a tradition as the most catholic of the metropolitan medical organisations, and, representing as it does all branches of the profession, its platform might well appear as highly appropriate to a wide and frank debate. Unfortunately on the present occasion there seems to have been no attempt to organise the discussion in advance, with the result that while a number of interesting speeches were made no contribution was forthcoming from the ranks of the general practitioner. In these circumstances it is not surprising that the discussion barely escaped from a somewhat narrow groove, and that with one or two limited exceptions it was concerned more with medical education than with medical practice. The consultants and the teachers have necessarily their share of interest in whatever concerns the welfare of their profession, but a verdict which does not include the evidence of the family practitioner hardly rests on a safe basis. A discussion conducted under these conditions is obviously incomplete, and the staff work responsible for it hardly merits commendation. In considering the opinions expressed it must then be remembered that the possibility of State regulation of the profession was excluded, and that no voice was heard from the practitioners who form the vast majority of the profession.

In dealing with the medical curriculum Colonel Waring, who opened the discussion, made a number of interesting suggestions, though he did not advance anything particularly novel. The addition of new burdens on the already labouring back of the student inevitably leads to a suggestion of a time extension of the curriculum, and this in turn has to be considered in its financial aspect. No one pretends that the average earnings of the practitioner are an abundant reward for the expense of his education, and if the charges in this respect are to be increased the supply of practitioners will be apt to decline, with obvious disadvantage to the public interest. Without question it is a matter for regret that the student so often leaves his school with but a scanty acquaintance with the more specialised branches of practice, and we entirely agree that he is compelled to spend too much time in listening to lectures which in not a few instances provide neither instruction nor inspiration. But even when the best is attained no curriculum which is at all possible will make the student a master in the whole of the medical art. All that can be gained is a foundation of sound principles, together with a scientific habit of mind and a capacity for exact observation. The application of these attainments to particular ends must be found in opportunities existing after the technical demands of the curriculum have been satisfied, and in actual life as well as in theory the practitioner's career must be conducted under the sense of the student spirit. An educational scheme which does not provide such opportunities will have no great worth, and the hope of greater efficiency will be much more likely to find realisation in this direction than in hurried courses in a crowded curriculum conducted under the shadow of an impending examination.

Some such conviction as that just expressed seems to have been present in the minds of several speakers in the recent discussion, as the necessity for the creation of enlarged post-graduation opportunities was generally allowed. The war has emphasised this necessity, for its close will mean the return of large numbers of men who have for various periods been detached from civil practice, and not a few of whom have no experience of such practice. Their recent experiences will have made some of them competent surgeons and some skilled administrators. But it will have done nothing to familiarise them with the general work of family practice or with those special forms of training which cannot be overtaken in the existing medical curriculum. Hence it is certain that there will be a considerable demand for clinical and other practical forms of training by men who in the technical sense are no longer students, seeing that they are already registered as qualified medical practitioners. There is still another consideration. Berlin and Vienna, the cities which formerly attracted practitioners from all parts of the world, are for many years practically closed to the English-speaking peoples, and the tide which used to set in these directions will find other outlets. Clearly in such circumstances the United Kingdom has a special opportunity to prove itself equal to the occasion. To London in particular the chance is an open and abounding one. Sentiment, facility of language, wealth of clinical material, and the halo of tradition combine to make it the centre to which medical practitioners from all parts of the British Empire and from the United States will naturally resort if only they can find here due provision for their educational needs. The chance is here and now, and it is in the highest degree unlikely that it will be renewed. Professor J. G. Adami, speaking on this point with the weight which arises from his professional eminence and from his special experience in Canada and in France, uttered a grave warning that unless London bestirs itself the stream will turn to other centres. It is all to the good that our French colleagues should be organising their great clinical resources and their capacity for effective and lucid teaching with a view to meet the certain demand for post-graduation work. They will, we doubt not, command, as they well deserve, great success. But this is no reason for hesitation and lethargy at home. Rather, indeed, it ought to be a stimulus to effort and enterprise, and to the determination that London shall take a worthy place in a rivalry that will be mutually beneficial. Hitherto it must be admitted that London has not succeeded in any scheme of organised post-graduation teaching. Where the blame lies it is not now necessary to inquire. The point of commanding importance is that there is now a unique opportunity of redressing this defect, and that if this opportunity is not seized the loss will be an enduring one. Both duty and advantage here point to one and the same claim, and the need for decision and achievement is pressing in the highest degree.

One point urged by Colonel Waring may be taken as evidence of a step in advance. He claimed that special arrangements, existing apart from those provided for medical students, must be made for practitioners, and no one rose to say him nay. The doctrine has not hitherto been generally accepted in the London medical schools, and pushed to its logical conclusion it seems to us to demand, as was claimed by one of the speakers, the reservation of one of the metropolitan hospitals for post-graduation instruction. Rumour has it that there is under discussion a scheme which would distribute practitioners engaged in post-graduation study among the existing medical schools with arrangements more or less specialised for their convenience. Our own opinion is decidedly in favour of the one hospital organised directly to the one end. Such a plan, of course, does not exclude work at other institutions, and some of these ought certainly to be linked with the central scheme. But really valuable clinical work means observation of cases not as isolated events but in continuity, and to run hither and thither to see this, that or the other great man is not the discipline which is really required. The student-practitioner needs an organisation specially devoted to his desires, and this must include a body of sustained clinical opportunity with which can be associated the whole of the teaching resources of the metropolis. A scheme which neglected these conditions came to a disastrous end shortly before the war, and a repetition of the experiment is not an inviting prospect. To some extent any plan must be experimental, for the exact wishes of the practitioners cannot yet be precisely estimated. But we are convinced that the principles here emphasised are vital, and we fear that if they are denied no large or worthy success can be anticipated. Though we write here particularly of London, we by no means forget the teaching value of other centres or of the numerous hospitals throughout the country. We have on more than one occasion advocated the worth of the latter as clinical organisations for the benefit of the practitioners in their neighbourhood, and such an enterprise seems to us one of the most important elements in any scheme of medical reconstruction. If London offers itself as a post-graduation centre especially attractive to practitioners from over the seas, the practitioner at home may reap at least an equal benefit from opportunities which are close to his hand, if only an enlightened policy will make these available. Post-graduation study has its special moments, but the best and most useful form of it is found in a continuous discipline.

